# Phytoliths as Structural, Physiological Integrators of Multifunctional Defense and Climate Resilience in Grasses

**DOI:** 10.3390/biology15211996

**Published:** 2026-11-04

**Authors:** Mudassir Ahmad Bhat, Neha Charak, Harshdev Singh, Sheikh Abdul Shakoor, Renu Bhardwaj, Ashok Kumar, Deepak Pathania, Fernanda Maria Policarpo Tonelli, Khalid Rehman Hakeem

**Affiliations:** 1 University Centre for Research and Development, Chandigarh University, Mohali 140413, Punjab, India; mabhat90@gmail.com (M.A.B.); renubhardwaj82@gmail.com (R.B.); 2 Department of Botany, Central University of Jammu, Rahya Suchani (Bagla) District Samba, Jammu 181143, Jammu and Kashmir, India; nehacharak2001@gmail.com (N.C.); harshdevsingh91@gmail.com (H.S.); ashok.bot@cujammu.ac.in (A.K.); 3 Department of Botany, Government Degree College Bijbehara, Anantnag 192124, Jammu and Kashmir, India; shakoorshiekh9@gmail.com; 4 Department of Environmental Sciences, Central University of Jammu, Rahya Suchani (Bagla) District Samba, Jammu 181143, Jammu and Kashmir, India; dpathania74@gmail.com; 5 Centro-Oeste Campus, Federal University of São João del Rei, Divinópolis 35501-296, Brazil; 6 Department of Biological Sciences, Faculty of Science, King Abdulaziz University, Jeddah 21589, Saudi Arabia; 7 Princess Dr. Najla Bint Saud Al-Saud Center for Excellence Research in Biotechnology, King Abdulaziz University, Jeddah 21589, Saudi Arabia

**Keywords:** phytolith, grasses, climate resilience, abiotic stress, biotic stress, combined stress, phytolith-occluded carbon (PhytOC), elevated CO_2_, silicon mode of action

## Abstract

Grasses (Poaceae) are the foremost silicon (Si) accumulators among terrestrial plants. Plants take up Si as monosilicic acid, Si(OH)_4_, which polymerizes in cells and tissues such as the epidermis, xylem and phloem to form phytogenic (biogenic) silica, SiO_2_ · *n*H_2_O, deposited as phytoliths. This review, based on a documented literature search with explicit inclusion and exclusion criteria, evaluates evidence that phytoliths protect grasses against herbivores, fungal and bacterial pathogens and nematodes, and against drought, heat, salinity, metal toxicity, UV-B radiation and mechanical stress, and it examines combined stresses under climate change. We distinguish phytolith-specific evidence from studies that supplied soluble Si or Si nanoparticles, and we grade the evidence for each proposed mechanism. Physical mechanisms (abrasion, mechanical and apoplastic barriers, reduced non-stomatal water loss) are the best supported. Biochemical and hormonal mechanisms, including jasmonic acid, salicylic acid and ethylene signaling, remain hypotheses because Si(OH)_4_ is chemically inert and much of the evidence is correlative. Elevated CO_2_ can lower foliar Si concentration in some grasses, which may weaken protection, but effects on total Si accumulation and function are poorly known. Phytolith-occluded carbon is a potential but uncertain carbon sink. We identify research priorities and practical implications for crop, pasture and carbon management.

## Simple Summary

Grasses absorb large amounts of silicon from the soil and store it in their cells and tissues as microscopic mineral particles called phytoliths. These particles act like natural armor against insects and grazing animals, and they may also help grasses cope with disease, drought, heat, salt, toxic metals and physical damage. They may also lock away carbon in soil for long periods. However, many studies have supplied dissolved silicon or silicon nanoparticles to plants instead of studying phytoliths themselves, and most have tested one stress at a time, whereas grasses in nature face several stresses together. The physical effects of the mineral are well supported, but claims that silicon triggers chemical or hormonal responses are much less certain, because dissolved silicon is chemically very unreactive. Rising carbon dioxide may also lower silicon concentrations in some grasses and weaken this protection. This review explains how the literature was searched, separates evidence on phytoliths from evidence on silicon supply, grades the evidence for each proposed mechanism, and argues that phytoliths should not be regarded as an all-purpose defense under future climates. These findings matter for predicting how grasslands and croplands will cope with climate change, and for food security and ecosystem health.

## 1. Introduction

### 1.1. General Introduction

Grasslands account for nearly two-fifths of the terrestrial ice-free land area and provide the basis for global food security through cereal and fodder production, but they are also among the ecosystems most vulnerable to anthropogenic climate change, including more frequent droughts and heat extremes, soil salinization, and shifts in the range of pests and pathogens [1,2]. Silicon (Si) is the second most abundant element in the Earth’s crust after oxygen, and grasses are among the most efficient Si accumulators in the plant kingdom, with foliar concentrations that commonly reach several percent and, in some species, about 10% of shoot dry mass, an order of magnitude more than in most dicotyledonous plants [3,4]. Plants take up Si as monosilicic (orthosilicic) acid, Si(OH)_4_ (also written H_4_SiO_4_), which polymerizes in the plant body to phytogenic silica, that is, amorphous hydrated biogenic silica (opal-A) with the general formula SiO_2_ · *n*H_2_O, deposited as microscopic mineral bodies known as phytoliths [5]. The condensation reaction can be written Si(OH)_4_ → SiO_2_ · *n*H_2_O + (2 − *n*) H_2_O. Phytoliths also occlude small amounts of organic carbon and trace elements [6], so SiO_2_ · *n*H_2_O describes the mineral phase and not the exact composition of the bodies. Throughout this review, “phytogenic silica” and “phytoliths” denote the solid phase, “Si(OH)_4_” denotes the dissolved monomer, and “Si” is used for the element or where the source studies did not specify the chemical form.

Phytoliths (from the Greek for “plant stones”) are microscopic bodies of amorphous hydrated silica (opal-A) that form when Si(OH)_4_ polymerizes inside or between plant cells [5,7]. Depending on the site of deposition, silica may fill the cell lumen, producing discrete bodies that take the shape of the cell; impregnate the cell wall; form a continuous layer within or beneath the cuticle; or accumulate in intercellular spaces and in the walls of trichomes, spines and the silicified cells of inflorescence bracts [8,9]. Because deposition follows cell shape, phytoliths retain the form of the cells in which they were produced, which makes them taxonomically informative [7,10,11,12,13].

In grasses, phytolith morphotypes fall into a small number of recognizable groups: silica short cells with diagnostic shapes (for example, bilobate, polylobate, cross, saddle, rondel and trapezoid forms), bulliform cells with a fan-like shape, silicified long cells with smooth, sinuous or dentate margins, and the silicified bases of trichomes, prickles and hairs [10,11,12]. Classification schemes, including the International Code for Phytolith Nomenclature, are described in Section 2.1. The frequency of morphotypes differs among grass subfamilies, which broadly reflects differences in photosynthetic pathway and habitat [10,11].

Four groups of functions are attributed to phytoliths in living plants: (i) physical defense against herbivores and pathogens, through abrasiveness, reduced digestibility and mechanical barriers; (ii) modification of water, ion and metal fluxes and of the optical and thermal properties of leaves under abiotic stress; (iii) structural support of leaves and culms; and (iv) long-term storage of silica and, within it, of a small fraction of organic carbon (phytolith-occluded carbon, PhytOC) in soils after plant death [4,6,14]. Outside plant physiology, phytoliths are widely used for archaeological and paleoecological reconstruction [7]; this use is not reviewed here.

Although the concept of phytoliths as a protective shield is widely cited, studies of defense against herbivores, tolerance of drought and toxic metals, paleoclimate reconstruction and the carbon cycle rarely refer to one another [14]. This matters because climate change alters many factors simultaneously: drought, heat, salinity, elevated CO_2_ and pest and pathogen pressure often co-occur, and plant responses to such combinations are frequently non-additive [2,15].

Silicon-mediated alleviation of stress has been reviewed repeatedly, including general syntheses [1,4,16], overviews of biotic and abiotic stress alleviation in crops [17,18,19], an overview for medicinal and aromatic plants [20], a quantitative meta-analysis of abiotic stress [21], and critical examinations of open questions in the field [22,23]. Most of these works are organized around individual crops or individual stresses and consider Si mainly as an applied supplement. The present review differs in four respects. First, it centers on the endogenous solid phase (phytogenic silica) in grasses, the group in which phytoliths are most abundant and best characterized. Second, it labels each body of evidence by what was actually manipulated or measured, so that effects of phytoliths are not conflated with effects of Si supply (see below). Third, it evaluates combined stresses and elevated CO_2_, which are largely absent from earlier syntheses, and it separates direct combined-stress experiments from hypotheses inferred from single-stress studies. Fourth, it includes an explicit critical evaluation of the mode of action of phytogenic silica (Section 9), which separates physical, physico-chemical and biochemical explanations and grades the evidence for each using stated criteria.

Evidence terminology and scope. The title and framework of this review concern phytoliths, but many of the cited studies supplied soluble silicate, Si fertilizer, foliar Si or Si nanoparticles to plants. Such studies show effects of Si supply, but they do not necessarily show effects of solid phytoliths. Throughout this review, evidence is therefore labeled by what was manipulated or measured. “Phytolith-specific” evidence (P) comes from studies that measured or manipulated deposited silica itself, for example, mandible wear on silica-rich versus silica-poor leaves, silica localization at infection sites, or PhytOC content. “Endogenous Si” evidence (E) comes from natural or genotypic variation in tissue Si concentration. “Si-supply” evidence (S) comes from experiments that added soluble silicate, Si nanoparticles or foliar Si and compared plants with low-Si or Si-free controls. Where a statement rests on S-type evidence only, it is worded as an effect of Si supply and not as a function of phytoliths. The evidence types are shown in Table 1, Table 2, Table 3 and Table 4 and in the figures. Following Section 1.2, “phytolith” and “phytogenic silica” denote the solid phase, “Si(OH)_4_” the dissolved monomer, and “Si” the element or the form unspecified in the source study.

This review therefore asks to what extent, and under what circumstances, phytoliths contribute to defense and climate resilience in grasses, and which of the proposed mechanisms are supported by direct evidence.

The question has practical consequences for agriculture and ecosystem management. First, Si fertilization and silicate amendments are increasingly proposed as a means of mitigating stress in cereals and pastures [17,19,24]. Whether the benefits arise from phytoliths, from soluble Si or from indirect effects determines how, when and at what dose Si should be applied, and whether benefits persist when supply stops. Second, genotypic variation in Si uptake, and the transporters that control it (Section 10.5), offer targets for breeding and genome editing, but only if higher Si accumulation reliably improves tolerance. Third, silica-based defenses reduce the digestibility of forage and may therefore reduce herbivore damage while also affecting livestock nutrition, which matters for grazing and pasture management [25,26,27]. Fourth, PhytOC links grass management to soil carbon storage, for instance through nitrogen fertilization and community composition [28,29], but its value as a net climate benefit depends on the uncertainties discussed in Section 8. Finally, if elevated CO_2_ lowers Si concentrations in some grasses [30,31], protection that is currently taken for granted in pest and drought management may decline under future climates.

### 1.2. Literature Search Strategy and Selection Criteria

This article is a narrative critical review supported by a structured and documented literature search. The search was planned and is reported following the transparency principles of the PRISMA 2020 statement (17). It is not a formal systematic review: no protocol was registered, individual studies were not scored for risk of bias, and no meta-analysis was performed. The purpose of the structured search was to make the selection of evidence reproducible and to reduce the risk of citing only studies that support the phytolith-shield hypothesis.

**Figure 1:**
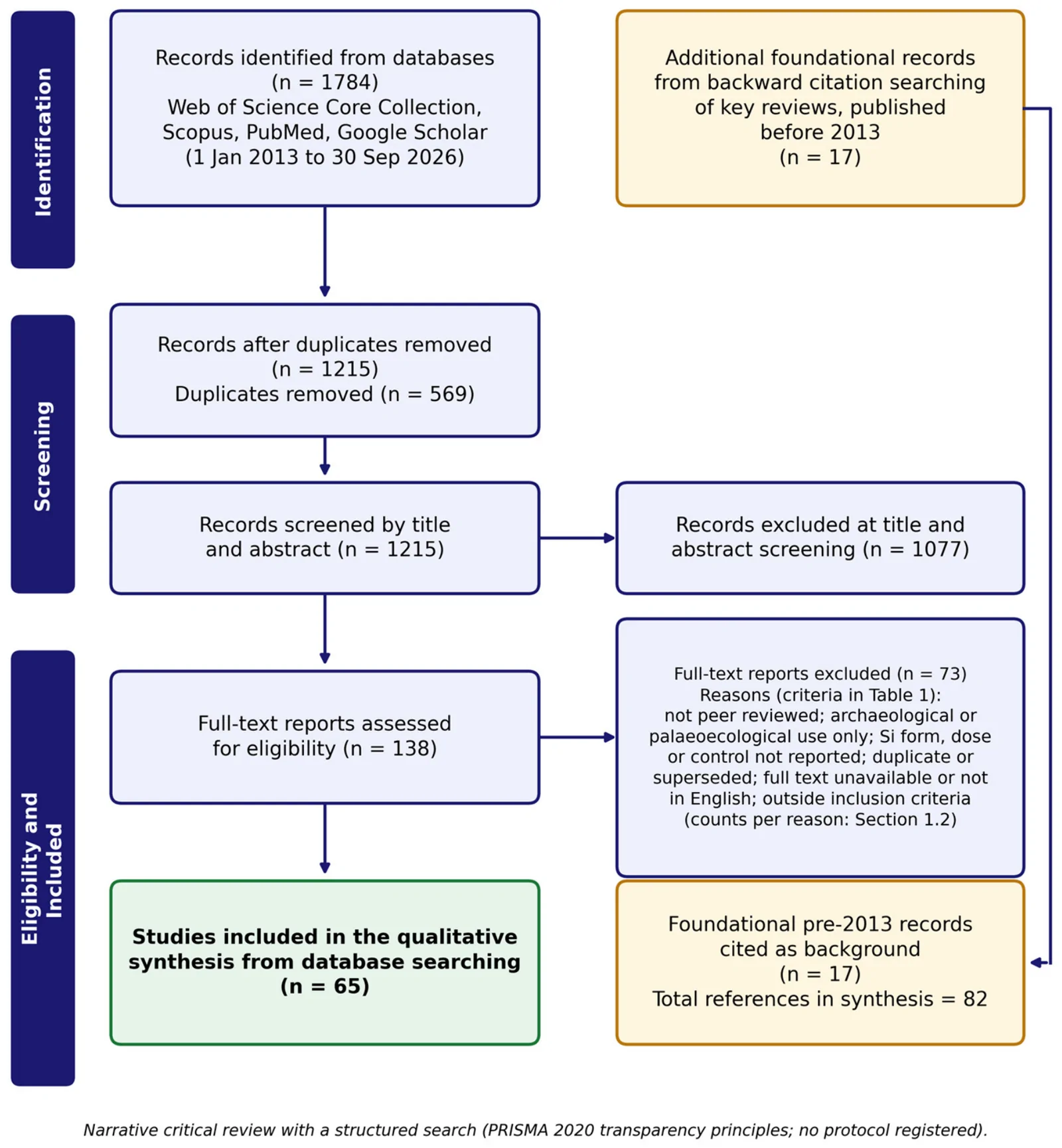
PRISMA 2020-style flow diagram of the literature search and study selection. The review is a narrative critical review supported by a structured search, and no protocol was registered.

Searches were run in the Web of Science Core Collection, Scopus and PubMed, and were supplemented with Google Scholar. The primary search window was 1 January 2013 to 30 September 2026. This window covers the period in which most of the mechanistic, meta-analytic and elevated-CO_2_ studies cited here were published [20,30,31,32,33]. Seminal records published before 2013 were not searched for systematically. Seventeen such records (foundational work on Si transport, phytolith classification, silica-based defense and PhytOC) were identified by backward citation searching of key reviews and are cited as conceptual, taxonomic and methodological background. They are counted separately from the 65 studies retained from the database searches (Figure 1). Title, abstract and author-keyword fields were searched in the first three databases. Because Google Scholar cannot be searched by field and ranks results by relevance, the first 200 records of each query were screened. Reference lists of key reviews [1,4,21,22,23] were searched backwards, and their citing articles forwards, to identify further studies. The complete database-specific search strings and the date on which each database was last searched are given in Appendix A.

The search used five concept blocks (Supplementary Table S1). Terms within a block were combined with OR; block A (the plant–Si system) was combined with each of blocks B (stress), C (mechanism) and D (climate and carbon) using AND; and every search was repeated with block E, a set of terms for null, negative or inconsistent outcomes, so that studies that do not support a protective role were deliberately sought.

Eligibility criteria are given in Supplementary Table S1. In brief, we included peer-reviewed primary studies, reviews, meta-analyses and academic book chapters, published in English, that addressed Si uptake, phytolith formation, or Si- or phytolith-mediated responses to defined stresses, with a preference for grass systems and for experiments that included an appropriate control. The chronological criterion was publication between 1 January 2013 and 30 September 2026 for records retrieved by database searching; pre-2013 records were eligible only as foundational background identified by citation searching, as described above. Studies of non-grass species were retained only when they provided mechanistic information not available for grasses, and they are identified as such in the text. We excluded conference abstracts, preprints, and these and other non-peer-reviewed sources: papers that used phytoliths solely for archaeological or paleoecological reconstruction (except where needed for classification); papers in which the Si form, dose or control treatment was not reported; and duplicate records.

Titles and abstracts, and subsequently full texts, were screened independently by two authors, and disagreements were resolved by discussion with a third author. The database searches identified 1784 records. After removal of 569 duplicates, 1215 records were screened by title and abstract, and 1077 were excluded at this stage. The full texts of 138 reports were assessed for eligibility, and 73 were excluded for the following reasons (numbers in brackets): non-peer-reviewed source (*n* = [6]); archaeological or paleoecological use of phytoliths without physiological, climate or carbon relevance (*n* = [14]); Si form, dose or control treatment not reported (*n* = [21]); duplicate or superseded report (*n* = [5]); full text unavailable or not in English (*n* = [8]); and non-grass system without mechanistic information that is unavailable for grasses (*n* = [19]). In total, 65 studies were included in the qualitative synthesis (Figure 1).

The literature on Si and plant stress is very large, and no review of this scope can be exhaustive. Where several studies addressed the same question, we prioritized (i) studies on grasses, (ii) primary experiments with a Si-free or low-Si control, (iii) meta-analyses and recent reviews, and (iv) studies reporting neutral or negative outcomes. The updated search also identified additional syntheses on Si and stress in crops and in medicinal and aromatic plants [10,11,12,13], which are cited in Section 1.1 and in the relevant sections. Studies that used soluble silicates, Si nanoparticles or foliar sprays are cited as evidence for effects of Si supply, but they are not treated as evidence for the action of phytoliths themselves (Section 9). Each study used as evidence was labeled by the form of Si (endogenous Si, phytogenic silica, soluble silicate, nanoparticles or foliar spray), the experimental system (hydroponic or other soilless culture, pot, growth chamber, glasshouse, field or cell culture), whether single or combined stresses were applied, and the species studied. These labels underlie the evidence types and confidence levels reported in Table 1, Table 2, Table 3, Table 4 and Table 5.

## 2. Phytolith Classification, Formation, Transport and Deposition in Grasses

### 2.1. Classification of Phytoliths in Grasses

Phytoliths have been classified in several ways, most commonly by morphotype. Grass phytoliths have been classified into four classes and 26 types [11]. The festucoid class has eight types (circular, rectangular, elliptical, acicular, crescent, circular crenate, oblong and oblong sinuous forms) and occurs mainly in cool-season C_3_ grasses of the Pooideae. The chloridoid class has two types (squat and thin saddle-shaped forms) and is found in C_4_ short grasses of arid habitats. The panicoid class has 11 types, including crosses with thick and thin shanks and dumbbells with short, long, straight or concave ends, and occurs in the Panicoideae and in warm-temperate C_4_ tall grasses. The elongate class comprises silicified long cells with smooth, sinuous, spiny or concave ends. This system was later extended to seven classes by adding the sasaoid class (short cells of bamboo), the fan-shaped class (bulliform cells) and the point-shaped class (prickle hairs) [12].

International Code for Phytolith Nomenclature (ICPN) 2.0 classification includes 19 morphotypes, including 12 general morphotypes, which consist of:

1. Spheroid psilate (Code: SPH_PSI): Solid or hollow phytoliths, resembling spherical to slightly ellipsoidal shape and devoid of ornamentation.
2. Spheroid echinate (SPH_ECH): Solid phytoliths having a spherical to slightly ellipsoidal shape and consisting of conical ornamentation over the surface.
3. Spheroid ornate: Solid phytoliths having complex ornamentation.

Other forms include Acute bulbosus, Blocky, Bulliform flabellate, Elongate entire, Elongate sinuate, Elongate dentate, Elongate dendritic, Papillate, and Tracheary and 7 Grass Silica Short Cell Phytoliths (GSSCPs), consisting of Saddle, Bilobate, Polylobate, Cross, Crenate, Rondel and Trapezoid [13]. Roughly around 110 morphotypes from grasses are currently known in total [34].

### 2.2. Phytolith Formation, Transport and Deposition in Grasses

Roots take up Si almost exclusively as uncharged monosilicic acid, Si(OH)_4_ (H_4_SiO_4_), which occurs at approximately 0.1–2.0 mM in soil solution [35]. Uptake and long-distance transport of Si involve two transporters first described in rice: Lsi1, an aquaporin-like influx channel in the exodermis and endodermis, and Lsi2, an active efflux transporter that enables Si to enter the xylem [36,37,38,39]. Because Si movement in the xylem is linked to transpiration, the amount of Si in an organ is related to the water lost from it by transpiration [5]. The Si cycle in plants and soils is also influenced by climate, and Si pools in paddy and upland soils depend on long-term weathering, plant uptake and recycling [40].

**Figure 2:**
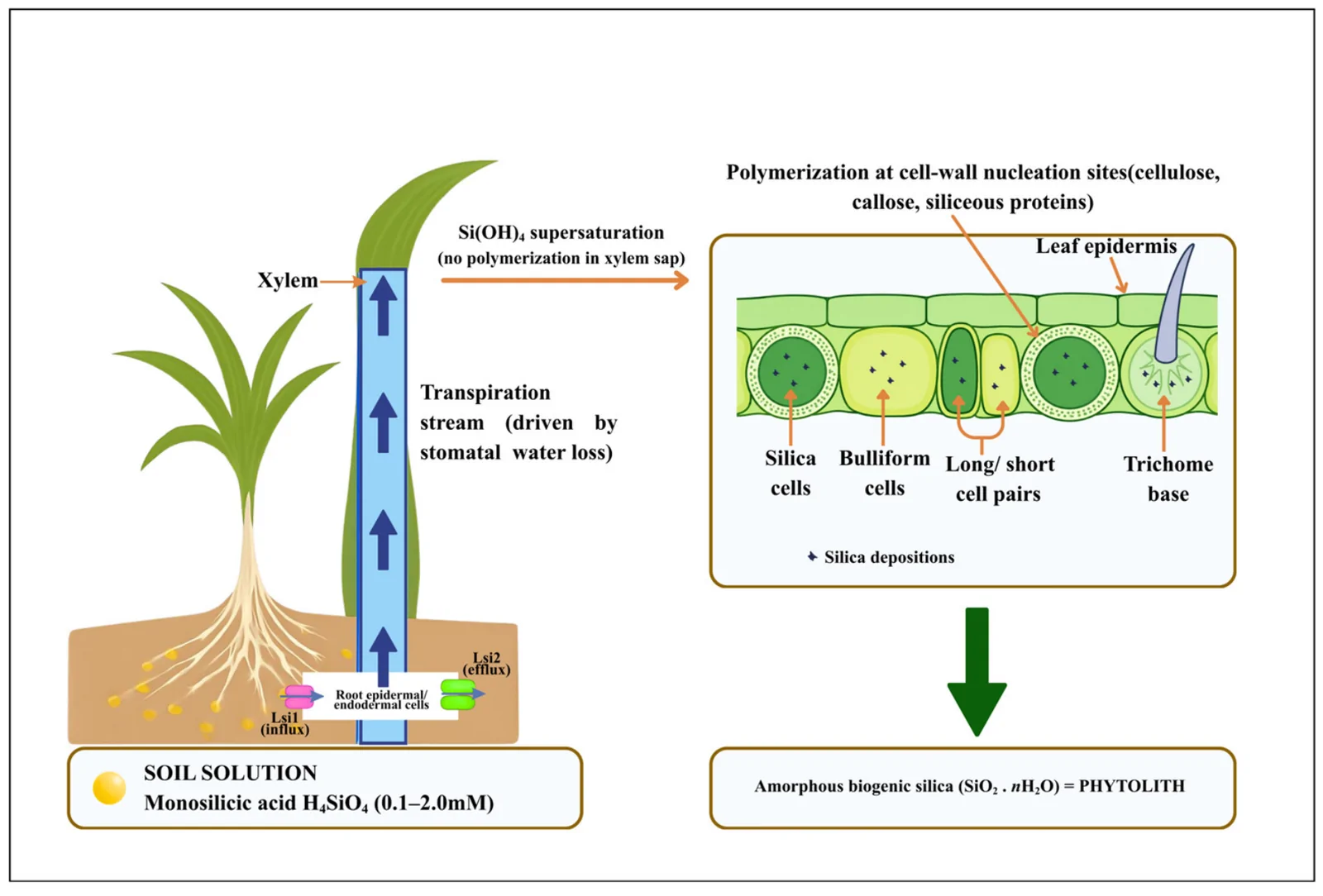
Silicon acquisition and phytolith formation in grasses. Si is taken up from the soil solution as uncharged monosilicic acid, Si(OH)_4_, through the influx channel Lsi1 and the efflux transporter Lsi2, travels in the transpiration stream, and, at supersaturation, condenses at cell-wall nucleation sites in the epidermis to form amorphous hydrated phytogenic silica, SiO_2_ · *n*H_2_O (phytoliths), according to Si(OH)_4_ → SiO_2_ · *n*H_2_O + (2 − *n*) H_2_O. Adapted from the principles described in [5,17,20].

In the xylem, Si(OH)_4_ remains monomeric even at concentrations about ten times above the saturation point for amorphous silica [39]. Conversion to solid silica occurs only when the sap reaches nucleation sites within the shoot [8]. The sites of deposition in grasses include silica short cells, silica–cork cell pairs, bulliform cells, trichome bases and a silica layer associated with the cuticle over the epidermis, which together give rise to the phytolith morphotypes that distinguish Poaceae taxonomically [8]. Deposition is not purely passive or driven by transpiration alone. Silicification occurs in tissues with low water movement and is blocked by metabolic inhibitors, which indicates that it is not simple evaporative concentration but a process that requires energy [8]. Histochemistry and elemental imaging further show that silicification is systematic and developmental rather than random [41], which is relevant to the observation that silicification induced by herbivores and pathogens can be rapid and spatially targeted. This view of Si transport and deposition draws on physiological and biochemical work [42,43], and it is now known to differ among plant lineages: grasses and other monocots are the most efficient Si accumulators, and taxa differ in their capacity for Si accumulation in ways that track phylogenetic relatedness and plant type [44]. Si accumulation is also shaped by evolution and biogeography. It has been proposed that grasses accumulated large amounts of Si during the Miocene, a period of low CO_2_ and seasonal aridity [14], and this hypothesis has since been tested experimentally [45]. Accumulation produces morphotypes specific enough to underpin paleobotanical phytolith analysis [30], a field that is not reviewed here. The processes described above are summarized in Figure 2.

## 3. Phytoliths as a Shield Against Herbivory: Constitutive and Induced Physical Defense

Defense against herbivores is the best-known function of grass phytoliths [27]. Silica-rich epidermal cells and silica-coated epidermis abrade the mandibles of insects and the teeth of mammals, and silica in leaf tissue makes digestion and nutrient extraction more difficult for the herbivore [25,26]. The cost to the herbivore accumulates with each feeding episode: mandible wear in insects is progressive and irreversible, so silica acts as more than a simple deterrent [26]. Silica-based defense can also be induced: silica deposition in grass leaves can rise above constitutive levels after repeated damage, and the response can differ among herbivore types [25,45,46]. Evidence for this physical effect comes mainly from studies that measured or manipulated silica in leaves (P- and E-type evidence) and is strongest for chewing insects; effects on sap-feeding insects are weaker (Section 4). Many of these experiments used potted plants under controlled conditions, and field data are scarce (Section 10.1).

### Silicon Deposition and General Stress-Related Responses

Reviews of Si and stress tolerance attribute a wide range of benefits to Si deposition, including protection against salinity, drought, radiation, lodging, hypoxia, heat, cold and metal toxicity [1,4,22]. Two physical explanations are most often proposed. First, silica deposited in or beneath the leaf cuticle is thought to reduce non-stomatal water loss under osmotic stress. Second, in roots, silica deposited around the exodermis and endodermis is thought to block the apoplastic bypass route and so restrict the movement of Na^+^, Cl^−^ and toxic metals to the shoot. The apoplastic obstruction hypothesis extends this idea by proposing that Si acts as an extracellular barrier that also interferes with pathogen effectors and haustoria, and so could affect both biotic and abiotic stress [22]. Studies that supplied Si to plants have also reported physiological, anatomical and molecular changes, which are summarized in reviews. In drought-stressed plants, Si supply has been associated with modified root anatomy, including endodermal silicification and suberization, and with better water uptake, chlorophyll retention and stomatal conductance [47]. In rice, Si supply was reported to upregulate stress-related genes such as *OsCMO* (choline monooxygenase) and *OsRAB16b*, which are involved in osmotic stress and dehydration responses [47]. Under salinity, Si supply has been associated with better preservation of chloroplast ultrastructure and with higher activities of the antioxidant enzymes superoxide dismutase (SOD), catalase (CAT), guaiacol peroxidase, ascorbate peroxidase (APX) and glutathione reductase [48]. Si supply has also been associated with greater synthesis of heat shock proteins and osmolytes and with reduced protein misfolding under heat [49]. All of these observations come from Si-supply studies (S-type evidence), and most are reported in reviews and not in primary studies. They show that Si supply is associated with these responses, not that phytoliths cause them. Whether the gene-expression and enzymatic changes reflect a direct action of Si or are secondary consequences of reduced stress damage is unresolved and is examined in Section 9.

## 4. Hormonal Regulation: The Jasmonic Acid Connection

A growing body of research connects Si-based defense to the jasmonic acid (JA) signaling pathway, which underlies many chemical defenses against chewing herbivores [32]. In rice, Si supply primed jasmonate-mediated anti-herbivore responses [32]. In the grass *Brachypodium distachyon*, Si deposition was triggered within six hours of applying methyl jasmonate, and JA signaling partly relieved the trade-off between Si-based and carbon-based (phenolic) defenses, so that both could be activated together [50]. Si supply was found to conserve hormonal defense signaling and to limit the growth of chewing herbivores but not to affect fluid-feeding insects [51], which suggests that the interaction is specific to feeding guild. Because aphids and other sap-sucking insects tend to activate the antagonistic salicylic acid (SA) pathway rather than JA, prior aphid feeding might be expected to weaken subsequent Si-based resistance to chewing herbivores. In *B. distachyon*, however, aphid feeding induced phytohormonal cross-talk without affecting Si defense against subsequent chewing herbivores [52], which indicates that the mechanism can be robust to mixed-guild attack in this system. Si supplementation and JA activation together increased phenolic defenses against a legume herbivore more than either treatment alone [53], so the synergy is not restricted to grasses. These studies are Si-supply experiments under controlled conditions. They show that Si supply and JA signaling interact, but they identify no molecular target through which Si(OH)_4_ could affect JA-dependent responses, and in several cases, the reported hormonal changes are associations observed after Si treatment and challenge, so cause and consequence cannot be separated (Section 9.3).

Below ground, Si supply increased root nodulation in a legume, which was associated with a higher abundance of herbivores [54], and Si has been proposed to influence defense against arthropod pests through both direct and indirect (tritrophic) effects [55]. In rice, Si supply altered the composition of herbivore-induced plant volatiles and increased the attraction of parasitoids to infested plants, which indirectly supports a role of Si in protection against herbivores [56]. Direct evidence for a below-ground Si–JA interaction against root herbivores is limited in the studies reviewed here. Together, these observations show that Si supply and JA signaling interact in some systems, but they do not identify the molecular target through which chemically inert Si(OH)_4_ could influence JA-dependent responses. Alternative explanations are evaluated in Section 9.3.

## 5. Pathogen Resistance: Fungi, Bacteria and Nematodes

Si supply has been associated with increased resistance to fungal and bacterial pathogens through both physical and biochemical routes [57]. A layer of silica beneath the cuticle can act as a mechanical barrier to the entry of fungal hyphae and appressoria [58]. Si supply also increases the activity of defense-related and oxidative enzymes, including peroxidase (POX), phenylalanine ammonia-lyase (PAL) and polyphenol oxidase (PPO) [59]. The rice–blast pathosystem (*Magnaporthe oryzae* and *M. grisea*) is the best studied. In this system, Si supply led to the formation of silica papillae and to cell-wall strengthening at sites of fungal entry [60], and it increased the accumulation of momilactone phytoalexins after infection, which suggests that physical protection is accompanied by biochemical defense [61] (Table 1). Most of this evidence comes from Si-supply experiments in rice under controlled conditions (S-type), and the biochemical component is correlative; it does not show that phytoliths themselves trigger the enzyme or phytoalexin responses.

**Figure 3:**
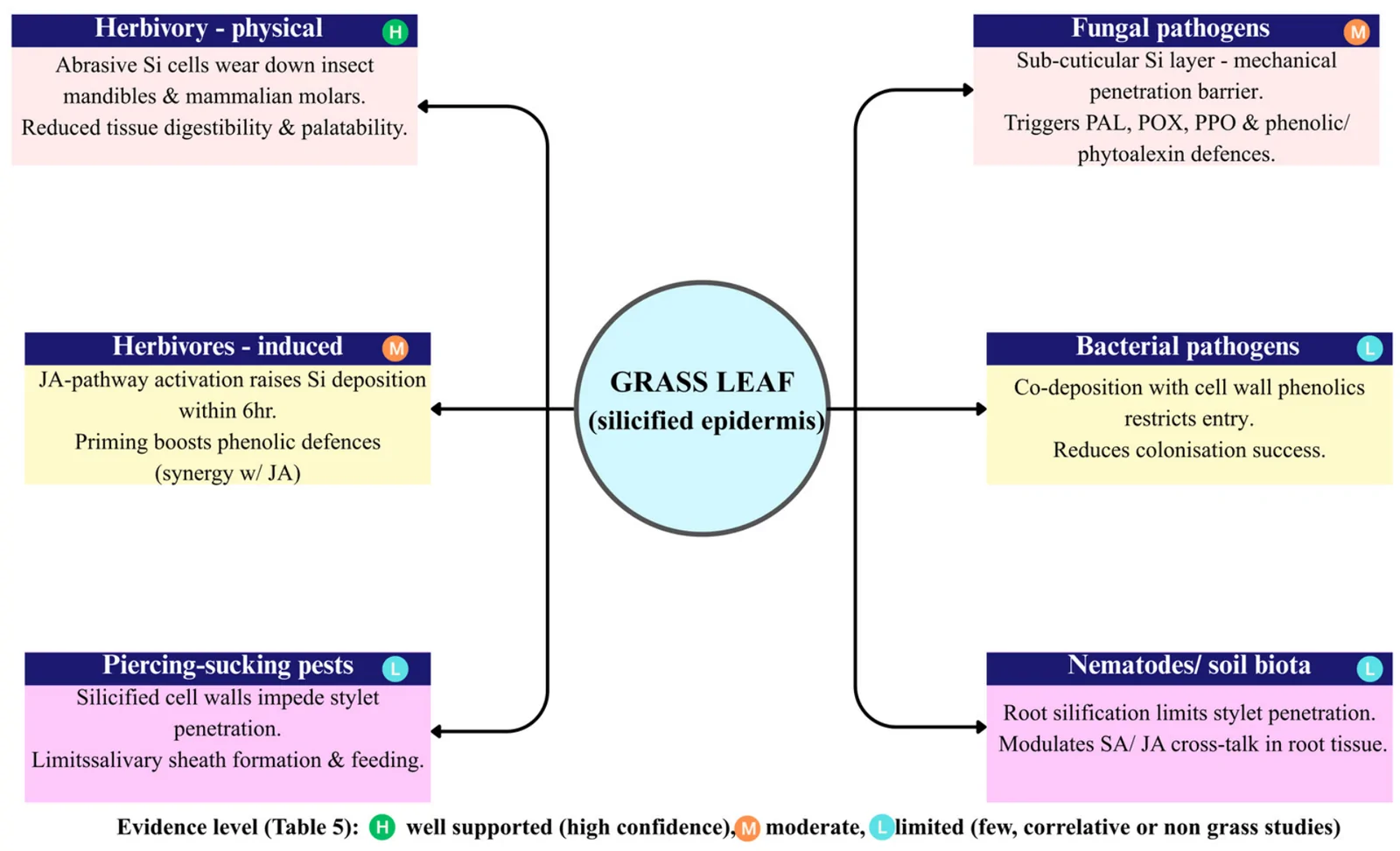
Convergent physical and biochemical mechanisms by which phytoliths and Si are proposed to defend grasses against herbivory, fungal and bacterial pathogens, piercing–sucking pests and nematodes [25,26,33,52,58,60,61]. Tags indicate the strength of evidence (criteria in Table 5): H, well supported; M, moderate; L, limited.. Evidence for the bacterial, nematode and piercing–sucking panels comes mainly from reviews and Si-supply studies, and the induced-defense and fungal panels rest largely on Si-supply experiments.

The effects of Si on disease resistance belong to the wider literature on mineral nutrition and plant disease [62]. They noted that Si-mediated disease control is not a single, universal process: Si may potentiate defense-gene induction against some biotrophic pathogens, but against necrotrophic pathogens such as *Cochliobolus miyabeanus* it may act independently of the JA/SA pathways [33]. The complexity of Si action implies that phytolith-based defense should be treated as a set of related, pathogen-specific mechanisms and not as a single framework [1]. In the rice *C. miyabeanus* system, Si-induced resistance was independent of the SA and JA pathways and was linked to interference with the pathogen’s exploitation of ethylene signaling [63]. Against nematodes, silicification of roots is proposed to reduce stylet penetration and to modulate SA–JA cross-talk in roots [1], but this proposal rests mainly on a review, and direct experimental evidence in grasses is limited (Figure 3). Section 9 evaluates these findings.

**Table 1:** Summary of phytolith- and Si-mediated mechanisms against major categories of biotic stress in grasses, with the type and strength of supporting evidence (P, phytolith-specific; E, endogenous Si; S, Si supply; see Section 1.1).

Biotic Stressor	Primary Mechanism (s)	Hormonal/Biochemical Link	Key Source (s)	Evidence Type and Strength
Chewing insect herbivores	Mandibular abrasion; reduced digestibility; progressive, cumulative fitness cost	Si–JA interaction reported; causal role unresolved	[26,51]	P/E and S; mostly controlled environments; physical effect well supported, hormonal link moderate
Mammalian grazers	Tooth wear; reduced palatability of silicified tissue	Silica deposition induced by repeated defoliation	[25,45,46]	E; induction shown in grasses; moderate
Piercing–sucking (phloem) feeders	Weak or no effect of silica; guild-specific response	SA-biased signaling; weaker Si effect than for chewers	[25,51,52]	E and S; consistent but few species; moderate (for a weak effect)
Root-feeding herbivores	Below-ground Si–JA interaction proposed; direct evidence limited	JA induction below ground (hypothesis)	[54,55]	S; indirect; limited
Fungal pathogens (biotrophic, e.g., rice blast)	Sub-cuticular mechanical barrier; papillae formation; phytoalexin induction	Potentiates SA pathway; momilactone induction	[58,60,61]	S; rice, controlled conditions; barrier moderate, biochemical link limited
Fungal pathogens (necrotrophic)	Interference with pathogen ethylene manipulation	JA/SA-independent route	[33,63]	S; single rice system; limited
Bacterial pathogens	Co-deposition with cell-wall phenolics proposed to restrict colonization	PAL/POX/PPO enzyme priming (associative)	[1]	Review-based; limited
Nematodes/soil biota	Root silicification proposed to limit stylet penetration	Root SA/JA cross-talk (proposed)	[1]	Review-based; limited
Natural enemies (3rd trophic level)	Altered HIPV blend increases parasitoid attraction	JA-mediated volatile induction	[56]	S; rice, single study; limited

## 6. Phytoliths, Silicon and Abiotic Stress Tolerance in Grasses

Most evidence on abiotic stress comes from experiments that supplied soluble Si or Si nanoparticles to plants in pots, hydroponics or growth chambers, and, for some stresses, from species outside the Poaceae. In this section, conclusions are restricted to the stresses and plant groups for which the cited studies give direct evidence, evidence from non-grass species is flagged, and statements about phytolith function are made only where the cited work addresses silica deposition itself. Field data are scarce, so effect sizes from controlled systems may not transfer to field conditions (Section 10.1).

### 6.1. Drought and Water Relations

Reviews and meta-analyses report that Si supply alleviates drought stress by maintaining leaf turgor, photosynthesis and water-use efficiency [21,23]. A silica–cuticle double layer is proposed to reduce cuticular transpiration [21,23], but direct measurements that separate this physical effect from the physiological effects of Si supply are scarce. Si supply is also associated with hormonal balance under drought, and with improved water use in wheat during drying phases [23]. A meta-analysis across many species found a consistent positive effect of Si addition on drought and several other abiotic stresses, but effect sizes were heterogeneous and depended on plant family and stress type [21]. It has likewise been questioned whether Si can be regarded as a panacea for drought and salinity [23]. Taken together, the evidence indicates that Si supply benefits drought tolerance in many experiments, through several effects, and that it is not a universal remedy.

### 6.2. Salinity

In salt-stressed plants, Si supply has been associated with reduced Na^+^ transport to the shoot, improved K^+^/Na^+^ selectivity and better maintenance of membrane and cell-wall function during osmotic stress, and apoplastic silica deposition is a proposed physical contributor [64,65]. In grasslands under high salinity, Si addition increased biomass carbon and phytolith-occluded carbon by supporting photosynthesis and membrane integrity [64], which links Si-mediated stress tolerance to the carbon storage discussed in Section 8. The evidence for a specific role of phytoliths, as opposed to Si supply, is indirect.

### 6.3. Heavy-Metal Toxicity

Si supply supports retention of toxic metals, and co-precipitation with silica and apoplastic deposition in cell walls are proposed to reduce metal movement to the symplast [4,66]. Reduced metal translocation was among the first benefits of Si to be recognized, and part of the chemistry has been demonstrated in cultured rice cells [67,68]. In experiments with Cd-contaminated rice and wheat, Si and Si nanoparticles reduced Cd transfer to shoots and grains and increased antioxidant enzyme activities under soil or foliar application [69,70], and foliar Si nanoparticles had similar effects in wheat under combined Cd and water-limited conditions [71]. Cd tolerance in rice also improved when Si oxide nanoparticles were combined with melatonin [72]. Si has been reported to reduce Cd and Cu toxicity in rice and to alter the expression of P-type toxic-metal ATPase and low-silicon (*Lsi*) genes and the levels of ABA, JA and SA [73], which suggests that metal detoxification and biotic defense may share hormonal networks; this is an inference from single-stress experiments (Section 7.2). A review summarizes Si effects on Cd stress through antioxidant activity, metal chelation and suppression of Cd transport genes [74]. Comparable benefits have been reported in non-grass species, including the common bean (*Phaseolus vulgaris*) [75]; such results show that the effect is not restricted to the Poaceae, but they cannot be used to infer grass-specific mechanisms. Most of these studies used pot or hydroponic systems with Si supply, so field relevance and the specific contribution of phytoliths remain uncertain.

### 6.4. Heat, UV-B and Mechanical Stress

**Figure 4:**
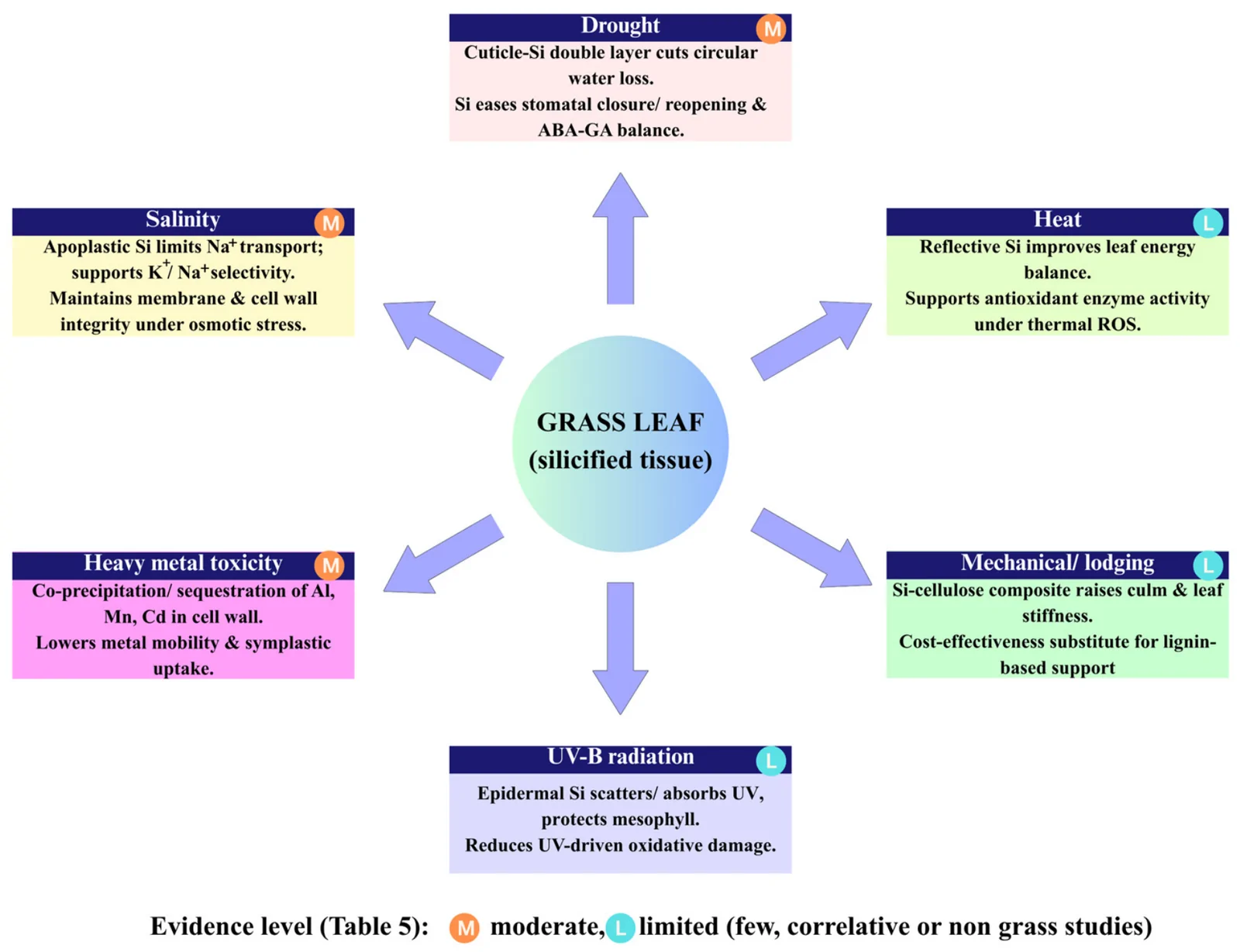
Proposed ways in which phytoliths and Si protect plants against abiotic stress (drought, heat, salinity, heavy-metal toxicity, UV-B radiation and mechanical stress). Tags indicate the strength of evidence (criteria in Table 5): M, moderate; L, limited. Evidence for heat comes only from a non-grass species. Synthesized from [1,4,14,21,23,64,65,66,69,70,71,76].

*Heat.* Si-associated improvements in leaf energy balance and antioxidant enzyme activity during heat-induced oxidative stress have been proposed [1], but direct evidence in grasses is very limited. The most detailed mechanistic study identified here was done in date palm (*Phoenix dactylifera*), a non-grass species, in which Si and gibberellin acted synergistically on ABA signaling and heat tolerance Figure 4 [76]. That study suggests that Si can modulate ABA-related signaling, analogous to the Si–JA interaction reported for herbivory, but it cannot be used to infer the mechanism in grasses.

*UV-B radiation.* Epidermal silica is proposed to scatter or absorb ultraviolet radiation and thereby protect the mesophyll from oxidative damage [1]. Direct experiments on this effect in grasses were not identified in the studies reviewed here, so the proposal remains largely hypothetical.

*Mechanical stress and lodging.* A composite of silica and cellulose in leaf blades and culms increases tissue rigidity and may partly replace lignin as a structural material [4,14]. Silica has been suggested to be mainly beneficial when carbon is limiting and habitats are arid [14], which would be consistent with the global expansion of grasses during the low-CO_2_ Miocene (Table 2). This is an evolutionary hypothesis supported by comparative and experimental work [14,30], and not a direct test of lodging resistance.

**Table 2:** Summary of Si- and phytolith-mediated mechanisms against major categories of abiotic stress in grasses (evidence from non-grass species is flagged), with the type and strength of supporting evidence (P, E and S as defined in Section 1.1).

Abiotic Stressor	Primary Mechanism (s)	Reported Outcome	Key Source (s)	Evidence Type and Strength
Drought	Proposed cuticle–silica double layer reduces water loss; hormonal rebalancing	Improved turgor, gas exchange and water-use of fficiency (Si supply)	[21,23]	S; meta-analysis, mostly controlled environments; moderate
Salinity	Apoplastic Si restricts Na^+^ transport; supports K^+^/Na^+^ selectivity	Higher biomass C accumulation in salt-affected grasses (Si supply)	[64,65]	S; grasses and other species; moderate
Toxic metals (Cd, Al, Mn, Cu)	Co-precipitation in cell wall; ATPase/Lsi gene regulation; hormone modulation	Reduced metal mobility, translocation and toxicity (mainly rice and wheat; Si supply)	[4,66,69,70,73]	S; rice and wheat, pot, hydroponic and cell-culture systems; moderate
Combined Cd + drought	Foliar Si nanoparticles under low soil moisture	Improved growth and reduced grain Cd under combined stress (wheat)	[77]	S; single controlled study; limited
Heat	Improved leaf energy balance and antioxidant support proposed; Si–GA synergy on ABA signaling	Reduced thermal oxidative damage (shown in date palm, a non-grass)	[1,76]	S; non-grass only; limited (hypothesis for grasses)
UV-B radiation	Epidermal silica proposed to scatter or absorb UV	Reduced mesophyll oxidative damage proposed	[1]	Review-based; no direct grass experiment identified; limited
Mechanical stress/lodging	Silica–cellulose composite proposed to increase stiffness	Possible substitute for lignin-based support	[4,14]	E; conceptual and comparative evidence; limited

## 7. Combined Biotic–Abiotic Stress: Synergies, Antagonisms and the Limits of the Shield

### 7.1. Why Combined Stress Cannot Be Predicted from Single-Stress Data

In nature, plants rarely experience one stress at a time, and responses to multifactorial stress cannot be predicted by adding responses to single stresses [2,15]. Some stress pairs interact synergistically. For example, drought and heat reinforce each other because heat increases transpirational water loss, whereas drought closes the stomata and raises leaf temperature. Other pairs interact antagonistically; for example, stomatal closure under drought can limit the entry of airborne stressors and of pathogens that infect through stomata. This non-additivity indicates that a phytolith-mediated or Si-mediated benefit measured under one stress cannot be assumed to persist, to be additive or to be enhanced when stresses co-occur. Physical effects of silica, such as abrasion and mechanical barriers, depend on the silica present at the site of attack and may therefore be more robust to stress combinations than effects that depend on hormone signaling, but this prediction has not been tested. In this section we therefore distinguish direct evidence from combined-stress experiments from hypotheses inferred from single-stress studies, and Table 3 labels each scenario accordingly.

### 7.2. Hormonal Cross-Talk and the Robustness of Si-Dependent Defense Under Combined Stress

The clearest combined-stress evidence linked to Si-dependent defense is the JA–SA cross-talk literature discussed in Section 4. Si-based defense against chewing herbivores depends strongly on JA, whereas sap-feeding insects and some abiotic stresses shift the balance toward the SA pathway. It is therefore theoretically possible that stresses inducing SA-related responses reduce the effectiveness of Si-dependent defense [51,52]. The only direct test identified here did not support this expectation: prior aphid feeding, which induced SA-related cross-talk, did not impair Si-dependent defense against subsequent chewing herbivores in *B. distachyon* [52]. This result comes from one species and one stress sequence and cannot be generalized. A second scenario is a hypothesis inferred from single-stress studies. Si-dependent Cd detoxification in rice involves ABA, JA and SA signaling [73], and these hormones are also central to defense against pests and pathogens. It has therefore been suggested that metal pollution and pest attack could compete for the same hormonal network, but no experiment identified here has combined metal and pest challenge, and this competition remains a hypothesis. The interactions in Table 3 are classified on the same basis, as either direct experimental evidence or hypothesis.

### 7.3. Elevated CO_2_ as a Potential Vulnerability of the Shield

Elevated atmospheric CO_2_ may be the climate-related factor most likely to weaken Si-based defense. Elevated CO_2_ and warming have been reported to affect Si-based and phenolic-based defenses differently in native and exotic grasses [31]. Tall fescue (*Festuca arundinacea*) was grown at CO_2_ concentrations typical of the Miocene (200 ppm), the present (410 ppm) and the Anthropocene (640 ppm) in controlled-environment chambers [30]. Elevated CO_2_ increased plant biomass and foliar carbon but lowered foliar Si concentration and antioxidant enzyme-based defenses, a pattern that suggests herbivores may perform better under high CO_2_ and low Si [30]. Three distinctions are needed to interpret these findings. First, a lower foliar Si concentration does not necessarily mean lower total Si accumulation: if biomass increases under elevated CO_2_, the concentration can fall through dilution even when the amount of Si per plant is unchanged or higher. Second, a lower concentration does not necessarily mean a smaller or less effective phytolith load, because defensive effectiveness depends on the amount, location and form of silica at the site of feeding or attack and not on bulk concentration alone. Third, defensive outcomes must be measured directly: a reduced Si concentration should be treated as a vulnerability only where it is accompanied by measured changes in total Si, silica distribution or herbivore and pathogen performance. Responses may also vary among species, environmental conditions and Si supply levels [30,31]; plants grown with ample soil Si might be affected less than plants with limited Si, but this has not been tested in the studies identified here. The effect of elevated CO_2_ on Si accumulation should therefore be viewed as an important potential vulnerability and not as a universal response.

Reduced CO_2_ was also used to test the evolutionary hypothesis that the phytolith defense of grasses evolved under the low CO_2_ of the Miocene, when carbon was limiting and conditions were seasonally arid [14,30]. Elevated CO_2_ also changes plant mineral composition more generally, so a reduced Si concentration may partly reflect altered mineral transport and dilution and not an effect specific to Si.

### 7.4. Combined Abiotic Stress and Silicon in Si-Accumulating Cereals

**Figure 5:**
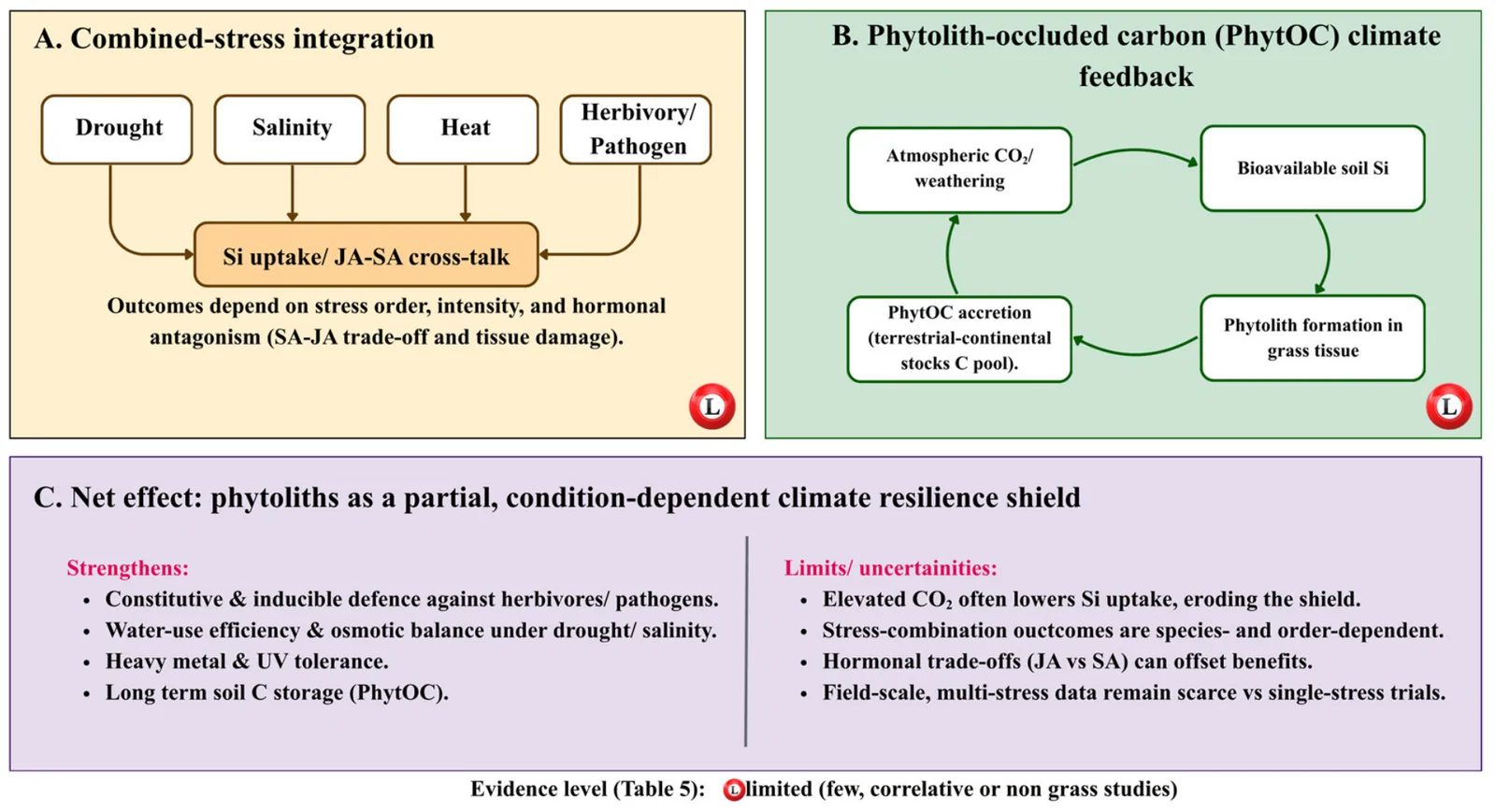
(**A**) Integration of multiple stresses on Si uptake and JA–SA hormonal interactions, as influenced by the order and intensity of stress; (**B**) the phytolith-occluded carbon (PhytOC) feedback loop; (**C**) a summary of the demonstrated effectiveness of the shield and its boundaries and uncertainties [2,14,15,30,57,71,73,79]. Tags indicate the strength of evidence (criteria in Table 5): panel (**A**) is largely inferred from single-stress studies (L), and panel (**B**) combines supported individual steps with an unquantified net effect (L).

Much of the empirical support for Si-mediated resilience under multiple stresses comes from experiments on Si-accumulating cereals. Foliar application of Si nanoparticles improved biomass and reduced grain Cd in wheat under combined Cd and drought stress in a controlled experiment [71]. In licorice (*Glycyrrhiza uralensis*), a non-grass species, Si combined with plant growth-promoting bacteria alleviated drought-induced oxidative stress [78]; this is a combination of treatments and not of stresses, and it is included only as an illustrative hypothesis for grasses. In all of these studies, Si was supplied externally to the soil or leaves or as nanoparticles, and none examined the in situ formation of phytoliths under naturally varying conditions (Figure 5).

**Table 3:** Illustrative combined-stress scenarios relevant to phytolith- and Si-mediated resilience in grasses. The last column distinguishes direct combined-stress experiments from hypotheses inferred from single-stress studies.

Stress Combination	Interaction Type	Implication for the Phytolith Shield	Key Source (s)	Basis of Evidence
Drought × heat	Synergistic (general plant stress physiology)	Whether Si or phytoliths modify this interaction was not tested in the studies reviewed (hypothesis)	[15]	General stress-combination literature; no Si-specific experiment; hypothesis
Chewing herbivory × prior aphid (SA-biasing) feeding	Largely independent in the tested case	Si–JA defense against chewers was not compromised by prior SA induction in *B. distachyon*	[52]	Direct experiment (Si supply, one species, controlled conditions)
Elevated CO_2_ × herbivory	Potentially antagonistic to the shield	Elevated CO_2_ lowered foliar Si concentration and some chemical defenses, in one study with greater herbivore performance; total Si and function not separated	[30,31]	Direct experiments (grasses, controlled environments); concentration-based
Cadmium × drought	Compounding (both stresses worsened growth without Si)	Foliar Si nanoparticles reduced grain Cd and improved growth under combined stress in wheat	[71]	Direct experiment (Si supply, wheat, single controlled study)
Cadmium × pest/pathogen hormonal demand	Potentially competing (shared ABA/JA/SA network)	Si-mediated metal detoxification involves the same hormone network as biotic defense; competition not tested	[73]	Inferred from single-stress studies; hypothesis
Drought × microbiome (PGPB)	Synergistic treatment effect (Si + bacteria)	Si and plant growth-promoting bacteria reduced drought oxidative stress more than either alone in licorice (non-grass); a treatment combination, not a stress combination	[78]	Direct experiment in a non-grass species; illustrative only

## 8. Phytoliths, Carbon Sequestration, and Climate Feedback

### 8.1. PhytOC Stocks and Their Management

Beyond protecting individual plants, phytoliths may contribute to carbon storage at the ecosystem level, because organic carbon (about 0.1–24.7% of phytolith mass, depending on species and plant part) is occluded during phytolith formation [6]. Occlusion within a silica structure is thought to shield this carbon from microbial decay, so that it may persist in soils for centuries to millennia and form a longer-lived fraction than most plant-derived carbon [6,79,80]. Grasslands are important in this context because grasses accumulate large amounts of Si and produce large quantities of phytoliths. In a survey of grasslands in China (79 sites, 237 soil profiles), the total PhytOC stock in soil was estimated at about 383 × 10^6^ tons of CO_2_ equivalent, with the Tibetan Plateau contributing more than 70% of the national total, and altitude and soil texture were the main controls on PhytOC distribution [79]. Further work suggests that below-ground PhytOC may exceed above-ground PhytOC [81]. At the species and community level, Si content, and therefore estimated PhytOC production, was higher in typical steppe than in meadow steppe in northern China [28], which links community composition to the grassland carbon sink.

This also has management significance: nitrogen application to degraded grassland in North China increased PhytOC production several-fold at moderate rates, but the effect declined at higher doses, which indicates that an optimum, and not a linear increase, in fertilization is needed to maximize phytolith carbon deposition [29].

### 8.2. Feedback Between Resilience Functions and Carbon Storage

The resilience functions described in Section 3, Section 4, Section 5 and Section 6 and the climate-mitigation potential discussed here are mechanistically linked, because both draw on the same Si pool. Silicate weathering and biogenic Si cycling control the supply of bioavailable Si in soil; grasses take up this Si and convert it into phytoliths, which contribute to the stress resilience described above; and part of the organic carbon allocated to phytoliths is occluded as PhytOC, which could provide long-term (centuries to millennia) removal of carbon from the active cycle [6,79]. If so, management that promotes phytolith production for resilience, such as silicate fertilization, could also increase long-term soil carbon stocks [64,79]. This feedback loop is potentially vulnerable to the reduction in Si concentration under elevated CO_2_ (Section 7.3). Combining the findings of the elevated-CO2 experiments on tall fescue [30] with the PhytOC literature suggests that elevated CO_2_ might weaken both phytolith-based defense and phytolith-based carbon storage, but this has not been tested experimentally.

### 8.3. Uncertainties in PhytOC as a Persistent Carbon Sink

The presence of organic carbon inside phytoliths does not by itself show that this carbon is a quantitatively important and persistent sink at ecosystem or global scales. Five uncertainties limit the strength of the climate-related conclusions. First, origin: PhytOC is derived from carbon fixed by the plant, so it represents a repartitioning of photosynthetic carbon into a more resistant fraction and not new carbon, and whether it constitutes net additional storage depends on how much of this carbon would otherwise have been mineralized. In addition, radiocarbon evidence suggests that some carbon in phytolith concentrates from grasses may derive from sources other than recent photosynthate, so analytical and source uncertainty in PhytOC measurements has not been fully resolved [82]. Second, stability: phytoliths are not inert, and their dissolution rates vary with phytolith properties, plant species and the chemistry of the surrounding solution [80]. Dissolution releases occluded carbon, and long residence times reported for particular soils or periods cannot be extrapolated to all environments [6,80]. Third, residence time: reported centennial-to-millennial persistence is based on a limited number of sites and methods, and the fraction of PhytOC lost through erosion, dissolution or turnover is poorly constrained. Fourth, stock versus sequestration: most published values are estimates of standing carbon stocks calculated from Si or phytolith content and PhytOC fractions, and they are not measurements of net, additional, long-term sequestration relative to a baseline. A stock estimate becomes a sequestration estimate only after production, turnover and loss are accounted for and, for management interventions, after the carbon costs of producing and applying fertilizers or amendments are subtracted. Fifth, scale and geography: much of the available stock data comes from grasslands in China [28,29,79,80], and extrapolation to other regions and to the global scale is not yet justified. For these reasons, PhytOC is best regarded as a potential component of ecosystem carbon storage whose magnitude remains uncertain, and not as an established climate-mitigation strategy.

## 9. Mode of Action of Phytogenic Silica: A Critical Evaluation

Section 3, Section 4, Section 5, Section 6 and Section 7 describe many physiological, biochemical and molecular changes that accompany Si supply or phytolith deposition. A balanced assessment must, however, separate two questions: whether Si is associated with greater stress tolerance, for which the evidence is extensive [19,20,21], and how Si acts, which remains contested [22,23]. The second question is difficult because of the chemistry of the element, which is considered first.

### 9.1. The Chemical Constraint: Si(OH)_4_ Is Biochemically Inert

Silicic acid is a very weak acid (p*K*_a_ ≈ 9.6–9.8), so at the pH of plant cell compartments, it exists as the neutral, tetrahedral molecule Si(OH)_4_ and does not ionize [5,83]. It has no redox chemistry under biological conditions, and no natural biochemistry is known in which it forms stable covalent bonds to carbon; Si–C bonds have been produced only by laboratory-evolved enzymes [84]. Plants are not known to possess Si-specific enzymes, cofactors, receptors or Si-containing metabolites, and Si is accordingly regarded as a beneficial but non-essential element [3,22,66]. Above about 2 mM at ambient temperature, Si(OH)_4_ is supersaturated with respect to amorphous silica and tends to condense, yet in xylem sap, it remains monomeric at concentrations well above this level until it reaches the sites of deposition [5,38].

These properties impose a logical constraint on the interpretation of the literature. If Si(OH)_4_ cannot take part directly in enzymatic or receptor-mediated reactions, any effect described as “biochemical” must be explained by (i) the physical presence of the solid phase, (ii) weak, reversible, non-covalent or hydroxyl-mediated interactions of Si(OH)_4_ or silica surfaces with organic molecules and metal ions, mainly in the apoplast, or (iii) indirect effects on the plant or on its pests and pathogens [22,83]. It follows that reports of altered gene expression, hormone levels or enzyme activities in Si-treated plants, however well documented, are not by themselves evidence for a direct biochemical role of Si.

### 9.2. Physical and Apoplastic Mechanisms: The Best-Supported Explanations

The strongest evidence concerns effects that need no biochemistry. Abrasive silica in leaves causes progressive, irreversible wear of insect mandibles and reduces digestibility [25,26], and the effect on chewing herbivores is well documented, although sap-feeding insects are much less affected [25,51]. Silica deposited beneath the cuticle and at sites of pathogen entry can act as a mechanical barrier [58,60], and apoplastic silica in roots can restrict the bypass flow of Na^+^ and toxic metals [64,65,66]. This view was formalized in the apoplastic obstruction hypothesis, in which Si accumulates in the apoplast and interferes physically and physico-chemically with the movement of effectors, ions and metals [22].

There is also evidence that Si becomes chemically associated with the cell wall. In cultured rice cells, most wall-bound Si was found in the hemicellulose fraction, with spectroscopic data consistent with Si–O–C cross-links, and this wall-bound Si was linked to reduced Cd uptake [67,68]. Si supply has also been reported to increase suberization and lignification in rice roots [77], which indicates that Si can alter wall composition. These data show that Si and cell-wall polymers interact, but they do not establish the chemical nature of the interaction in intact tissues, whether it is causal for tolerance, or how it would be sensed by the plant. Physical explanations also have limits. They predict that benefit should scale with the amount and location of deposited silica, yet Si-associated resistance to powdery mildew has been reported in *Arabidopsis thaliana* [85,86], a species that accumulates little Si [44], which suggests that barrier effects alone are insufficient.

### 9.3. Evidence for Biochemical and Hormonal Involvement, and Its Limitations

A second body of work links Si to hormone signaling and defense metabolism. In rice, Si supply primed jasmonate-mediated anti-herbivore responses [32], and Si and JA can act synergistically on defense in grasses and a legume [50,51,53,59]. Si has also been reported to modulate ABA, JA and SA levels and the expression of metal transporter genes under Cd stress [73]. Several limitations, however, prevent these observations from being read as evidence for a direct biochemical action.

First, the effects are specific to species and pathosystems. Si-induced resistance of rice to *C. miyabeanus* operated independently of SA, JA and ABA and was associated with interference with the pathogen’s exploitation of ethylene signaling, and not with activation of host immunity [63], and Si-associated resistance of *Arabidopsis* to powdery mildew involved mechanisms other than the SA pathway [86]. Second, most effects are observed only after challenge and are consistent with priming, that is, a stronger response to stress, and not with signaling by Si itself; the molecular basis of priming by Si remains unknown, and the chemical mechanisms of Si effects on hormonal defense have been described as indirect and poorly characterized [32,59]. Third, many reported changes are downstream of stress severity. If Si reduces tissue damage or Na^+^ and metal uptake by physical means, lower reactive oxygen species, weaker induction of antioxidant enzymes and altered hormone levels are expected as secondary consequences. A meta-analysis found that Si consistently reduced oxidative damage in stressed plants but did not consistently increase antioxidant production [21], which is hard to reconcile with Si acting as a direct elicitor of antioxidant defenses. Without damage-matched comparisons, this “less stress” signature cannot be distinguished from Si-induced signaling.

Fourth, experimental design often confounds Si with other variables and differs from field conditions. Comparisons are commonly made between Si-supplemented plants and plants grown in Si-free media, an artificial condition that may itself cause Si-deficiency effects [15]; soluble silicates change the pH of the medium and add K^+^ or Na^+^; and Si nanoparticles and foliar sprays have poorly characterized dissolution and uptake [69,71]. Many studies were also done in hydroponic or other soilless systems and in pots under controlled conditions, where Si availability, root environment, stress intensity and exposure duration differ from those in the field, so results may not transfer to field conditions [21,23]. Fifth, no Si receptor, sensor or Si-binding protein with a demonstrated signaling role has been identified, and the genetic tools that exist, such as rice *lsi1* and *lsi2* mutants, have been used mainly to study uptake and not signaling [38]. Finally, most mechanistic work comes from a small number of Si accumulators (rice, wheat, *Brachypodium*), and Si transport and accumulation differ among lineages [44]; generalization across the Poaceae is therefore unwarranted at present.

### 9.4. Hypotheses That Can Reconcile Inert Chemistry with Observed Responses

Several non-exclusive hypotheses can reconcile a chemically inert precursor with biologically active outcomes (Table 4). Physical reinforcement and obstruction require no biochemistry and are the most parsimonious explanation for herbivore and many abiotic effects. Physico-chemical interactions in the apoplast, including binding of Si to hemicellulose and phenolics, co-precipitation with metals and adsorption of ions, could alter wall properties and ion fluxes [22,67,68]. Altered wall mechanics or integrity could in principle be perceived by damage- and wall-integrity-sensing systems that, in other contexts, are coupled to JA signaling; this idea would explain how Si could influence JA-dependent responses without a Si-binding receptor, but it is speculative and has not been tested for Si. Priming, in which prior Si supply changes the amplitude of stress responses by unknown means, is supported by the rice and grass data [32,59]. Si may also interfere directly with pest or pathogen biology, for example, with pathogen-derived ethylene [63], with effector delivery [22] or through altered herbivore-induced volatiles [56]. Finally, Si may act indirectly through nutrition, water relations or the rhizosphere microbiome [49,78]. Table 4 summarizes the supporting evidence, main limitations and confidence for each hypothesis, graded with the criteria in Table 5. These criteria are adapted from the general considerations of the GRADE approach to rating evidence [81]: the number and independence of studies, the directness of the evidence (phytolith-specific, endogenous Si or Si supply; grass or non-grass; single or combined stress), study design and controls, consistency among studies, and demonstration of causality. The grading is a structured qualitative appraisal by the authors and not a formal GRADE assessment.

**Table 4:** Proposed modes of action of phytogenic silica and Si in grasses, with the supporting evidence (P, phytolith-specific; E, endogenous Si; S, Si supply), main limitations and the authors’ confidence in each, based on the criteria in Table 5.

Proposed Mode of Action	Supporting Evidence (Examples)	Main Limitations	Confidence (Authors’ Appraisal)
Physical abrasion and mechanical barrier	P/E: progressive mandibular wear and reduced digestibility [25,26]. S: sub-cuticular barriers and papillae in rice [58,60]	Guild-specific, weak against sap feeders; costs to the plant not fully quantified; mostly controlled-environment experiments	High for chewing herbivores; moderate for pathogens
Reduced non-stomatal water loss	S: silica–cuticle layer proposed; meta-analysis of abiotic stress [21,23]	Effect sizes heterogeneous and dependent on plant family and stress type [21]; direct measurements separating physical from physiological effects are scarce	Moderate
Apoplastic obstruction and physico-chemical binding	S: restricted Na^+^ and metal bypass flow [64,65,66]; Si bound to hemicellulose and reduced Cd uptake in rice cells [67,68]; altered suberization and lignification [85]	Mostly rice roots and cell cultures; chemistry of binding and causal role *in planta* unresolved	Moderate
Hormonal priming (JA, SA, ABA)	S: JA priming in rice [32]; Si–JA synergy [50,51,59]; hormone changes under Cd stress [73]	Species- and pathosystem-specific; SA/JA-independent cases [63]; molecular target unknown; may be secondary to reduced damage	Moderate for herbivory in some grasses; low elsewhere
Interference with pest or pathogen biology	S: pathogen ethylene manipulation [63]; altered herbivore-induced volatiles [56]	Few pathosystems; not shown for most grasses	Low to moderate
Direct antioxidant and gene-expression effects	S: numerous supplementation studies [47,48]	Oxidative damage consistently reduced but antioxidants not consistently increased [21]; Si-free controls; changes may be downstream of stress severity	Low
Indirect effects via nutrition, water status or microbiome	S: rhizosphere reconfiguration [49]; Si with plant growth-promoting bacteria in a non-grass species [78]	Emerging evidence; causality untested	Low
Wall-integrity or mechanical sensing (hypothesis)	None specific to Si; inferred from other systems	Untested	Very low (hypothesis)

**Table 5:** Criteria used to assign confidence levels to the proposed mechanisms in Table 4. Combined-stress claims are capped at low confidence unless supported by a direct combined-stress experiment.

Confidence Level	Criteria
High	Direct (P or E) evidence from several independent studies in at least two species or systems; consistent direction of effect; appropriate controls; causal link supported by manipulation (for example, blocking or varying silica deposition) or by measurements at the site of action.
Moderate	Several studies, but restricted to a few species or based mainly on S-type evidence; direction of effect generally consistent; plausible mechanism partly tested; causal link not fully demonstrated.
Low	One or few studies, inconsistent results, or only correlative evidence (associations after Si supply); evidence from reviews or non-grass species only; claims about combined stress without a direct combined-stress experiment.
Very low (hypothesis)	No direct test; inferred from other systems or from chemical plausibility.

### 9.5. Consequences for the Phytolith-Shield Concept and Testable Predictions

Taken together, the evidence supports the phytolith shield as a heuristic that unites physical and physico-chemical protection at the tissue level; it does not yet support the shield as a unified biochemical mechanism. Three consequences follow. First, claims about signaling should be framed as hypotheses unless damage-matched comparisons are provided. Second, studies should distinguish the solid phase (phytoliths and silica deposits) from soluble Si(OH)_4_ and from engineered forms such as nanoparticles, because physical mechanisms depend on the solid phase, whereas indirect chemical effects may depend on Si(OH)_4_ supply. Third, the hypotheses in Table 4 make different predictions. Physical explanations predict effects that scale with silica content at the site of attack and that disappear if deposition is blocked, whereas signaling or priming explanations predict effects that persist after Si is withdrawn and that remain at equal levels of damage. Suitable approaches include *lsi1* and *lsi2* mutants and other lines with contrasting Si accumulation, silicate controls matched for pH and cations, time-resolved hormone and transcript profiling within hours of challenge, and high-resolution mapping of Si at feeding and infection sites [8].

## 10. Critical Appraisal: Contradictions, Gaps, and Future Directions

### 10.1. The Single-Stress Bias

Most of the mechanistic evidence presented above comes from studies that test one stressor at a time, usually in controlled conditions such as glasshouses, growth chambers, pots and hydroponic or other soilless systems. This is a valid way to establish mechanisms, but it is a weak basis for predicting stress interactions in the field, for three reasons. First, controlled systems differ from field conditions in Si availability, root environment, stress intensity and duration, and the frequency of fluctuating or sequential stresses, so effect sizes measured in pots or soilless culture may differ from those in the field [21,23]. Second, as shown in Section 7, only a few experiments have combined stresses directly [30,31,52,71], and the hormonal cross-talk and stress-combination scenarios discussed in Section 7.1 and Section 7.2 are largely inferred from single-stress data. Third, many Si studies, whether of single or combined stresses, measured the effect of Si supply on biochemical processes without examining phytolith formation, so their results cannot be attributed to phytoliths without further evidence (Section 9). Future work should therefore use factorial designs that combine at least two stresses at realistic intensities, include Si-free or low-Si and pH-matched controls, measure silica deposition directly, and extend from pots to multi-season field trials.

### 10.2. Elevated CO_2_ as an Underappreciated Vulnerability

The finding that elevated CO_2_ lowers foliar Si concentration and, in some experiments, Si-associated defenses [30,31] sits uneasily with the characterization of phytoliths as a “climate-resilient” trait, and it deserves more attention than it currently receives in the Si literature. A more nuanced interpretation is that the phytolith barrier protects against some environmental pressures (heat, drought, salinity, pest attack) but is itself vulnerable to others (rising CO_2_). Because the available evidence is limited to a few species and to concentration-based measurements (Section 7.3), the effect should be presented as an important potential vulnerability and not as a universal response. Very few studies have tested Si-based protection under elevated CO_2_ combined with another environmental stress, and few, if any, have separated effects on tissue Si concentration, total Si accumulation and functional outcomes; this is a major gap exposed by this review.

### 10.3. Mechanistic Pluralism Versus a Unified “Shield” Narrative

Si acts through multiple, non-overlapping pathways in biotrophic and necrotrophic pathogens, and its effects differ between chewing and phloem-feeding herbivores [33,51]. Metal detoxification involves ATPases and hormones [73]. Together, these findings suggest that “the phytolith shield” cannot be regarded as a universal mechanism applicable to all stressors. Some reviews present Si as a broadly acting solution to many problems, which overstates the unity of what is mechanistically a set of related but stress-specific processes; critical syntheses caution against this view [22,23]. The evaluation in Section 9 reinforces this point: the physical and physico-chemical mechanisms are better supported than the biochemical ones, and the evidence for signaling roles should be regarded as hypothesis-generating.

### 10.4. Taxonomic and Methodological Gaps

Most information on the mechanism of Si–JA signaling comes from studies on a handful of model or crop grass species (notably *B. distachyon*, rice and wheat), whereas most information on PhytOC and carbon sequestration comes from grassland systems in China [28,29,79,80]. Testing these findings in a wider phylogenetic and biogeographical range of grasses, including the C_4_ grasslands of Africa and the Americas, would show whether current results reflect global trends or regional and taxonomic peculiarities. Reliance on pot experiments in which Si was applied as nanoparticles, particularly in studies of toxic metals [69,70,75], is a further limitation.

### 10.5. Genetic Engineering of Silicon Transporter Genes to Enhance Environmental Stress Tolerance

The key Si transporters identified are Low silicon 1 (Lsi1), a channel-type influx transporter, and Low silicon 2 (Lsi2), an energy-dependent efflux transporter, which together mediate root uptake and xylem loading of silicic acid [39]. Homologues of these genes have been characterized in rice (*Oryza sativa*), barley (*Hordeum vulgare*) and wheat (*Triticum aestivum*) [5,87]. Lsi1 and Lsi2 are therefore candidate targets for manipulating Si uptake and phytolith formation in economically important cereals. Overexpression of Lsi1 and Lsi2, or CRISPR-based editing of their promoters to strengthen stress-responsive expression, could increase Si deposition. Such approaches would, however, need rigorous testing, because higher Si accumulation does not translate uniformly into higher tolerance (Section 7 and Section 9). Loss-of-function lines such as *lsi1* and *lsi2* mutants also provide genetic tools for testing the mode of action of Si (Section 9.5).

### 10.6. Research Priorities

*Priority 1: field and multi-stress experiments.* Multi-year field trials that apply realistic combinations of drought, heat and herbivory to grasses, and that measure endogenous phytolith formation and not only exogenous Si addition, are needed to close the gap between controlled-environment and field evidence. Experiments that test Si-based protection under elevated CO_2_ combined with other stresses, extending the design of the tall fescue experiments [30] to a wider range of grass species and stress environments, should separate effects on tissue Si concentration, total Si accumulation, silica distribution and functional outcomes.

*Priority 2: carbon and stress-specific mechanisms.* Field measurements of PhytOC formation and persistence in regions beyond China, including Africa, the Americas and Australia, would allow a clearer evaluation of current carbon-sequestration estimates, and they should distinguish standing stocks from net sequestration and quantify dissolution and loss. Si action against necrotrophic pathogens and leaf chewers should be compared in the same experimental setups to test whether defense is one mechanism or several. Phytolith production and Si addition should be compared in alleviating toxic-metal stress, including Cd combined with drought, in cereals grown in the field. Finally, the proportion of variation in Si accumulation that is attributable to environmental and genetic factors should be quantified to establish whether phytolith production is fixed or adaptable.

*Priority 3: mode of action.* The mode of action of phytogenic silica should be tested by (i) combining *lsi1*/*lsi2* mutants or other lines with contrasting Si accumulation with Si-free, Si-supplemented and pH- and cation-matched control treatments; (ii) comparing Si-treated and control plants at equal levels of tissue damage to separate direct signaling from secondary effects; (iii) time-resolved hormone and transcript profiling within the first hours after challenge; (iv) high-resolution localization of Si at infection and feeding sites; and (v) comparing soluble Si(OH)_4_, solid phytogenic silica and characterized Si nanoparticles.

## 11. Conclusions

Phytoliths give grasses multifunctional but not universal protection. The best-supported functions are physical: abrasion and reduced digestibility for chewing herbivores, mechanical and apoplastic barriers, and reduced non-stomatal water loss. Evidence for hormonal and biochemical roles is weaker, and because much of the literature concerns Si supply and not phytoliths, many claims remain hypotheses (Section 9.4 and Section 9.5).

*Potential applications.* In agriculture, the evidence supports using Si management mainly where physical protection is relevant, for example, against chewing insects in cereal and pasture systems, where silicate amendments or selection of high-Si genotypes could reduce damage, and as one component of integrated management of drought, salinity and metal-contaminated soils, with the dose, form and soil context optimized locally. Pasture and grazing management may exploit induced silica defenses, but the effect of silica on forage digestibility must be weighed against its protective benefits. Candidate targets for breeding and genome editing include the Si transporters Lsi1 and Lsi2, provided that higher Si accumulation is shown to improve tolerance in the target environment (Section 10.5). At the ecosystem scale, phytoliths link grass community composition and nutrient management to soil Si and carbon cycling, and grassland management that increases phytolith production could contribute to soil carbon storage, although this requires verification.

*Limitations of current research.* The evidence base is dominated by controlled-environment experiments on a few model species (rice, wheat and *Brachypodium*) by studies of Si supply and not of phytoliths, by single-stress designs, and by PhytOC estimates from a limited geographic range. Evidence on heat, UV-B, bacterial and nematode defense, and on combined stresses is particularly thin, and the effect of elevated CO_2_ on total Si accumulation and function is unknown.

*Future directions.* The most promising priorities are (i) multi-year field trials with factorial stress treatments that measure phytolith formation directly; (ii) mutants and contrasting genotypes with matched controls to test whether Si acts through physical, physico-chemical or signaling routes; (iii) experiments under elevated CO_2_ that separate Si concentration, total Si and function; and (iv) PhytOC studies that distinguish standing stocks from net, long-term sequestration across more regions (Section 10.6). Until such evidence is available, phytolith-based protection should be regarded as climate-resilient only conditionally: it is effective against several single stresses, but its effectiveness under combined stress and elevated CO_2_ is unknown and may be diminished.

## Data Availability

No new data were created or analyzed in this study. Data sharing is not applicable to this article. The complete database-specific search strings are provided in Appendix A.

## Supplementary Materials

The following supporting information can be downloaded at: https://www.mdpi.com/article/10.3390/biology15211996/s1, Supplementary Table S1: Literature search strategy and eligibility criteria used in this review. Database-specific search strings and search dates are given in Appendix A.

## Appendix A. Database-Specific Search Strings and Search Dates

The search blocks below were combined as described in Section 1.2 and Supplementary Table S1 (A AND B, A AND C and A AND D, each repeated with block E). Strings were adapted to the syntax of each database; truncation () was applied to single words, and quoted phrases were used for multi-word terms.

Block A: (silicon OR silica OR “silicic acid” OR phytolith OR “biogenic silica” OR silicification) AND (grass OR Poaceae OR cereal OR rice OR wheat OR maize OR barley OR millet OR bamboo OR Brachypodium)

Block B: herbivor OR pathogen OR nematode OR “biotic stress” OR “abiotic stress” OR drought OR saline OR “toxic metal” OR cadmium OR heat OR “UV-B” OR lodging OR “stress combination” OR “multifactorial stress” OR “combined stress”

Block C: “mode of action” OR mechanism OR “jasmonic acid” OR “salicylic acid” OR “abscisic acid” OR ethylene OR priming OR transcriptome OR “gene expression” OR “cell wall” OR apoplast OR biochem

Block D: “elevated CO2” OR “carbon dioxide” OR “climate change” OR “carbon sequestration” OR PhytOC OR “phytolith-occluded carbon”

Block E: “no effect” OR “no significant” OR “did not” OR negative OR “trade-off” OR inconsistent OR controvers OR critique

**Table A1:** Database-specific search syntax and date of the last search.

Database	Search String (Blocks A–E Are Defined in Supplementary Table S1)	Date Last Searched
Web of Science Core Collection	TS = (A) AND TS = (B); TS = (A) AND TS = (C); TS = (A) AND TS = (D); each repeated with AND TS = (E). Limits: publication years 2013–2026; language English.	[30 September 2026]
Scopus	TITLE-ABS-KEY (A) AND TITLE-ABS-KEY (B); with C and D in place of B; each repeated with AND TITLE-ABS-KEY (E). Limits: PUBYEAR > 2012 AND PUBYEAR < 2027; LANGUAGE (English).	[30 September 2026]
PubMed	(A)[tiab] AND (B)[tiab]; with C and D in place of B; each repeated with AND (E)[tiab]. Limits: publication date 1 January 2013 to 30 September 2026; English.	[30 September 2026]
Google Scholar (supplementary)	Plain-text versions of A AND B, A AND C and A AND D (and with E); first 200 records of each query screened.	[30 September 2026]
