# Supplementary material for: Phytoliths as Structural, Physiological Integrators of Multifunctional Defense and Climate Resilience in Grasses
Source: Biology (Basel). 2026 Nov 4;15(21):1996. doi: 10.3390/biology15211996 (PMC13761500; doi:10.3390/biology15211996)
Supplement: Supplementary file 1 [file biology-15-01996-s001.zip › biology-4613501-supplymentary.pdf]

Supplementary Table S1. Literature search strategy and eligibility criteria used in this review. Database-specific search strings and search dates are given in Appendix A.

Item	Details
Databases	Web of Science Core Collection; Scopus; PubMed; Google Scholar (supplementary)
Period, language and search dates	Primary window: 1 January 2013 to 30 September 2026; English-language records. Foundational pre-2013 records were added by backward citation searching. Date last searched by database: Appendix A.
Search fields	Title, abstract and author keywords (WoS: TS; Scopus: TITLE-ABS-KEY; PubMed: [tiab]); Google Scholar: first 200 records per query
Block A (plant–Si system)	(silicon OR silica OR "silicic acid" OR phytolith OR "biogenic silica" OR silicification) AND (grass OR Poaceae OR cereal OR rice OR wheat OR maize OR barley OR millet OR bamboo OR *Brachypodium*)
Block B (stress)	herbivor OR pathogen OR nematode OR "biotic stress" OR "abiotic stress" OR drought OR salin OR "toxic metal" OR cadmium OR heat OR "UV-B" OR lodging OR "stress combination" OR "multifactorial stress" OR "combined stress"
Block C (mechanism)	"mode of action" OR mechanism OR "jasmonic acid" OR "salicylic acid" OR "abscisic acid" OR ethylene OR priming OR transcriptome OR "gene expression" OR "cell wall" OR apoplast OR biochem
Block D (climate and carbon)	"elevated CO2" OR "carbon dioxide" OR "climate change" OR "carbon sequestration" OR PhytOC OR "phytolith-occluded carbon"
Block E (null or negative outcomes)	"no effect" OR "no significant" OR "did not" OR negative OR "trade-off" OR inconsistent OR controvers OR critique
Combination	A AND B; A AND C; A AND D; each repeated with AND E
Inclusion criteria	(i) Peer-reviewed original research, reviews, meta-analyses and academic book chapters; (ii) Poaceae as study system, or non-grass system giving mechanistic information not available for grasses (flagged in the text); (iii) addresses Si uptake and transport, phytolith formation, Si- or phytolith-mediated responses to a defined biotic or abiotic stress, stress combinations, elevated CO2 or PhytOC; (iv) Si-treated versus control plants, or contrasting endogenous Si, with stress and Si form and dose reported
Exclusion criteria	(i) Conference abstracts, preprints, these and other non-peer-reviewed sources; (ii) archaeological or paleoecological phytolith studies without physiological, climate or carbon relevance (used only for classification); (iii) Si form, dose or control not reported; (iv) duplicate or superseded records; (v) full text unavailable or not in English
Screening	Two authors independently screened titles/abstracts and then full texts; disagreements resolved by a third author
Records	Identified from databases n = 1784; foundational pre-2013 records from citation searching n = 17; duplicates removed n = 569; screened n = 1215; excluded at title/abstract n = 1077; full texts assessed n = 138; excluded n = 73 (reasons in Section 1.2); included n = 65 (Figure 1).
